# Integration of Adenylate Kinase 1 with Its Peptide Conformational Imprint

**DOI:** 10.3390/ijms23126521

**Published:** 2022-06-10

**Authors:** Cheng-Hsin Wu, Chung-Yin Lin, Tzu-Chieh Lin, Dar-Fu Tai

**Affiliations:** 1 Department of Chemistry, National Dong Hwa University, Hualien 97403, Taiwan; m9812022@gms.ndhu.edu.tw (C.-H.W.); m9812012@gms.ndhu.edu.tw (T.-C.L.); 2 Medical Imaging Research Center, Institute for Radiological Research, Chang Gung University, Taoyuan 33302, Taiwan; 3 Department of Nephrology and Clinical Poison Center, Chang Gung Memorial Hospital, Taoyuan 33302, Taiwan

**Keywords:** adenylate kinase 1, molecularly imprinted polymers, conformation, secondary structure, inhibition

## Abstract

In the present study, molecularly imprinted polymers (MIPs) were used as a tool to grasp a targeted α-helix or β-sheet of protein. During the fabrication of the hinge-mediated MIPs, elegant cavities took shape in a special solvent on quartz crystal microbalance (QCM) chips. The cavities, which were complementary to the protein secondary structure, acted as a peptide conformational imprint (PCI) for adenylate kinase 1 (AK1). We established a promising strategy to examine the binding affinities of human AK1 in conformational dynamics using the peptide-imprinting method. Moreover, when bound to AK1, PCIs are able to gain stability and tend to maintain higher catalytic activities than free AK1. Such designed fixations not only act on hinges as accelerators; some are also inhibitors. One example of PCI inhibition of AK1 catalytic activity takes place when PCI integrates with an AK1_9-23_ β-sheet. In addition, conformation ties, a general MIP method derived from random-coil AK1_133-144_ in buffer/acetonitrile, are also inhibitors. The inhibition may be due to the need for this peptide to execute conformational transition during catalysis.

## 1. Introduction

There is a growing interest in and need for artificial enzymes [1,2]. Although researchers have explored many experimental and computational methods for elucidating the chemical mechanisms of enzymes, most of these sophisticated methods are for noninvasive observation [3]. To study the catalytically competent state of an enzyme, researchers routinely use a substrate or inhibitor as an interloper to bind the active site [3]. Notable exceptions to this reliance on substrates and inhibitors involve the use of mechanical force or miniaturized tweezers [4,5]. A recent discussion about the relationship between conformational dynamics and catalysis aroused our interest in adenylate kinase 1 (AK1, EC 2.7.4.3) [6,7,8].

AK1 coordinates the major signaling pathways directly responsible for adenosine triphosphate (ATP) production [9], assisting in the production of satisfactory responses to a diverse range of functional, environmental, and stress stimuli, as needed for cells to thrive [10]. Substantial evidence suggests that AK1 plays a central role in bioenergetic regulation, most notably that of ATP levels [11,12]. Dysregulated bioenergetics leading to altered cellular bioenergetics and mitochondrial function [13] is a major feature of several human diseases, including diabetes and hypertrophic cardiomyopathy [14], metabolic disorders [11], cancer [15,16], and neurodegenerative diseases [17,18]. An ideal modularity has yet to be established, but it could help in the early diagnosis of a range of diverse diseases or in designing AK enzymes as valuable targets for innovative treatments. In addition, neither structural nor biochemical approaches can be applied in the conformational transition during catalysis owing to the blockade of the kinetic properties between AK segments and different ligands.

**Figure 1:**
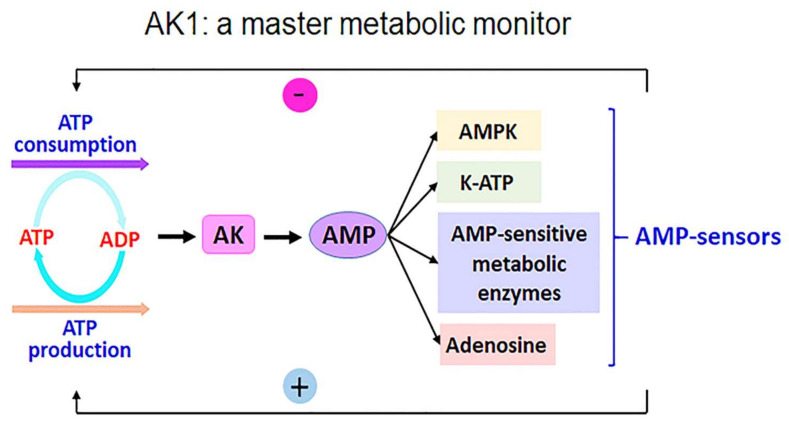
Adenlyate kinase (AK) enzyme activity. AK enzymes are often used as a metabolic monitor of energy load, leading to the activation or inhibition of downstream enzymes [11]. ATP, adenosine triphosphate; ADP, adenosine diphosphate; AMP, adenosine monophosphate; AMPK, AMP-activated protein kinase; K-ATP, ATP-sensitive potassium channel.

In this regard, a special point of discussion has focused on the closing and opening conformational transitions that characterize conformational dynamics during catalysis [19,20]. AK catalyzes the reversible phosphoryl transfer reaction ATP + AMP ↔ 2 ADP, maintaining the cellular energy balance between AMP and ADP/ATP (Figure 1) [11,21]. The AK isoform (AK1 and AK2) network senses ATP/ADP via the cell and cellular compartments and delivers energy phosphoryl groups of ATP to ATPases [11]. Significant progress has been made in defining the dynamics of conformational transitions that are functionally important in AK catalysis.

Conceptually, enzymes probably use hinged motion to significantly lower the activation free energies of reactions before the enzymes bind to a substrate [22,23]. So far, there has been no direct experimental evidence pointing to the dynamic contributions of conformational changes to AK1 catalysis. A tool for verifying the calculations of enzyme dynamics would be very useful. As AK1 is a flexible enzyme [24,25], it undergoes extensive conformational changes during catalysis [26]. The conformation of an enzyme can be altered by exploiting organic solvent interactions [27]. Previously, several AK1 peptides were reported to transform from random coils to secondary structures after the introduction of special solvents [28,29]. Mixtures of buffer and 2,2,2-trifluoroethanol (TFE) or methanol are essential to this type of transformation when it involves linear peptides.

Despite the randomly organized structure of molecularly imprinted polymers (MIPs) and their unpredictable formulating mechanism, their ability to recognize target molecules has been reported repeatedly [30,31]. Research has shown that the imprinting of random-coil peptides can successfully generate the desired nano-cavities for the corresponding peptides and proteins [32]; however, the relative structure between peptide templates and their mother protein is still ambiguous [33]. To broaden the scope of our method, we turned our attention to forming cavities for α-helix and β-sheet peptides.

In the present study, we introduce a general method for constructing a peptide conformational imprint (PCI) in a special solvent in order to modify enzyme catalysis, where the forming cavities can mechanically grasp a hinged region of the given enzyme. We also present a new and entirely unexplored mechanism for inhibition or activation based on sequence recognition. This phenomenon may be termed ”conformation-tightening”, and synthetic copolymers can be used as a ”conformation tie”, generated using either a PCI process or a general MIP method without any special solvent [33,34]. During the enzyme capture in our experiments, this conformation tie produced conformation freezing (or fixation) at a part of the enzyme that could not be envisaged directly in previous studies. A mechanical trigger such as the conformation tie permits the manipulation of enzyme activity.

## 2. Results and Discussion

**Figure 2:**
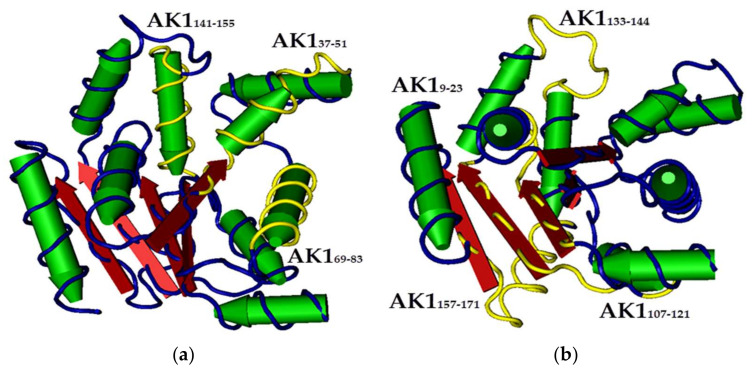
The structure of adenylate kinase 1. (**a**) α-helixes; (**b**) β-sheets and random coil. Yellow: selected sequence.

In this study, we (1) used MIPs to induce the stabilization fixation of protein secondary structure and (2) analyzed the recognition and catalytic activity of AK1. Screening of hinges [35] able to influence catalysis upon capture was undertaken. As shown in Figure 2a, three AK1 peptides with an α-helix secondary structure (37–51, 69–83, and 141–155) were selected. Figure 2b shows the locations of three peptides with β-sheet secondary structures (9–23, 107–121, and 157–171) and one peptide with a random-coil structure (133–144). Using circular dichroism (CD), previous research [28,29] has verified that these peptides possess α-helix or β-sheet structures in various mixtures of buffer and TFE.

**Figure 3:**
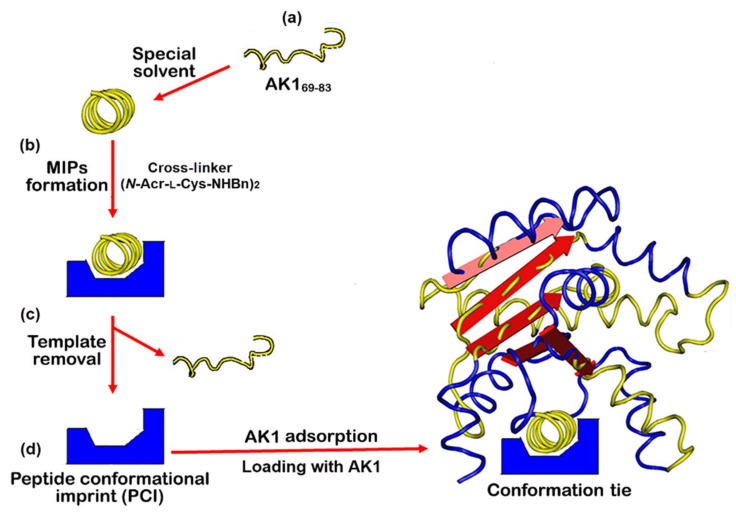
The fabrication of a peptide conformational imprint for adenylate kinase 1 (AK1). (**a**) Preparation of the AK1_69-83_ as a template. (**b**) Fabrication of hinge-mediated molecularly imprinted polymers (MIPs) in a special solvent using cross-linker (*N*-Acr-L-Cys-NHBn)_2_ and the template. (**c**) After template removal, the peptide conformational imprint (PCI) was complementary to the template peptide. (**d**) PCI loading with AK1. The recognition of an AK1 and its characteristic fragments as acting as an accelerator or an inhibitor is a conformation tie.

Using MIP technology, the selected AK1 peptide segments were synthesized as templates to generate α-helix and β-sheet cavities. The AK1 recognition sites, imprinted with peptide segments, were established using two types of solvent systems during the polymerization. Random-coil cavities were fabricated in the mixture of acetonitrile/buffer (AB), as previously described [33,36]; PCIs were constructed similarly but in the presence of a special solvent. Figure 3 presents an example of PCI fabrication.

As we expected and as the CD spectrum confirmed, the binding affinity of PCIs, when formed in a special solvent, corresponded to the secondary structure of the PCIs’ template. Our results clearly demonstrate how the special solvent alters the conformation of peptides. As shown in Table 1, the random-coil cavities constructed in AB were able to bind their templates by using AB as the mobile phase. Higher K_d_ values were measured while testing the MIP-grafted chips with a mixture of MeOH/buffer (MB) for the mobile phase. In contrast, PCIs constructed in a special solvent possess memory, and thus PCIs bind their template when they use MB as the mobile phase, indicating that an α-helix or β-sheet cavity has been generated. We observed that the resonant frequencies using the MIP-based QCM sensing system associated with the AB were lower than those associated with the MB.

The TFE/buffer proved to be a better special solvent system than the MB, with the former exhibiting a higher boiling point and generating a set of more precise cavities. Nevertheless, MB is a more convenient and economical mobile phase. As the ratio of peptide conformations in the special solvent is unique, the resulting PCIs have unique proportions of random-coil, α-helix, or β-sheet cavities. Therefore, the K_d_ values of these PCI-grafted chips can be optimized with different solvent ratios. Interestingly, AK1_107-121_ can be transformed from a random-coil form to a β-sheet form and, further, to an α-helix form.

There is a web of direct relationships between defects in AK1, AMP metabolic signaling, and human diseases [11,14,15,16,17,18]. Thus, in the present study, the PCI-grafted chips were tested for their ability to detect human AK1 (21.64 kDa MW, isoelectric point 9.24; 194 amino acids). To demonstrate the binding effect of the PCIs on chips, previous studies have examined the AK1 dissociation constants of PCI-QCM chips [37,38]. We performed a QCM assay and detected nanogram amounts of target AK1 on different PCI chips, confirming that the chosen peptides could serve as templates for the fabrication of PCIs. Furthermore, the chosen 15-mer of the AK1 segments were long enough to mimic the secondary structure of natural AK1 in a special solvent.

To evaluate the conformational transitions of enzymes, several PCIs were used as the enzyme conformation ties. Whether on an α-helix or a β-sheet, the binding effect of the PCI-AK1s proved workable (Table 2). Attractive interactions occurred when the protein and the cavity were conformationally matched. The K_d_ value of the TFE-induced α-helix cavities ranged from 40 to 200 pM, figures that are generally better than those associated with random-coil cavities generated in an AB. Therefore, these results have important implications for the development of improved PCIs capable of enhancing protein capture. Ultimately, one can further optimize the K_d_ value of the PCI chip for AK1 by using a different ratio of special solvent. Although the affinities are generally poor for a MB system, the K_d_ value of the AK1_107-121_Cp_7M3B_ reached 50 pM when the MB served as the special solvent at a 7:3 ratio.

AK1 catalyzes the phosphorylation of AMP by ATP to form two ADP molecules. So that their catalytic activity in solution can be evaluated, AK1 has to be constrained and quantified. PCIs were grafted onto QCM chips, which adsorbed AK1 and resulted in QCM resonant frequency shifts near 20 Hz. We assayed the PCIs for their catalytic activity [39]. We conducted quantitative assays of ATP, ADP, and AMP in the presence of PCI-AK1s, with all measurements corresponding to peak areas of the chromatogram.

Previously, enzyme inhibition has been reported with regard to templates in which MIPs used whole protein [40,41]. Therefore, we were expecting the hinge region to exhibit a reduced rate of reaction, and when, upon capture, the PCIs would require an expansion of movement, the head and tail would probably move further, while the core region would move less. However, the direct experimental evidence from our study points toward the occurrence of a dynamic conformational transformation during AK1 catalysis. In fact, some of the PCI-AK1s demonstrated even better catalytic activity than the free enzymes did. This phenomenon was demonstrated upon capture at the α-helixes (AK1_37-51_, AK1_69-83_, AK1_141-155_) and the β-sheets (AK1_107-121_, AK1_157-171_). When the AK1 segment was chosen in the hinge region and AK1 underwent conformational transformation, the PCIs maintained their same secondary structures. Capture not only maintains stability but also minimizes energy loss during the catalytic process, thus accelerating catalytic activity [34,35].

**Figure 4:**
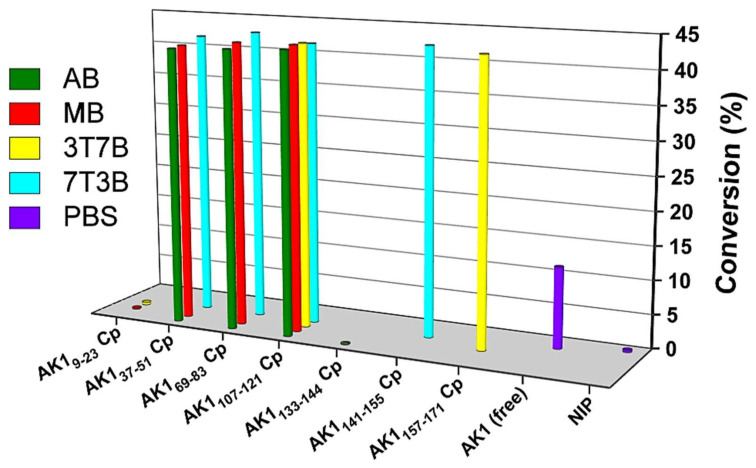
The effect of integrating a conformation tie into AK1’s catalytic activity. Cp, chip; NIP, non-imprinted polymer. Buffer: pH 7.2, 20 mM PBS buffer; AB, CH_3_CN/buffer = 1:1; MB, CH_3_OH/buffer = 1:1; 3T7B, TFE/buffer = 3:7; 7T3B, TFE/buffer = 7:3.

As shown in Figure 4, the observed activation of PCI-AK1s varied little, indicating that the amount of AK1 that integrated into the PCIs was similar to the free AK1. Although each PCI contained a unique proportion of random-coil, α-helix, or β-sheet cavities, only active AK1 was tightly bound to a conformationally matched cavity. Notably, consecutively dipping PCI-AK1s (AK1_37-51_, AK1_69-83_, AK1_107-121_, AK1_141-155_, and AK1_157-171_) with fresh AMP and ATP solution resulted in an obviously greater conversion rate than for the free AK1 activity. The results show that PCI-AK1s can be used to further enhance AK1 activity in the conversion of phosphorylation of AMP by ATP.

We observed two types of inhibition during our examination of the catalytic reaction. First, one of the chosen peptides, AK1_9~23_, was in the hinge region [35], and its PCI_9~23_ turned out to be an inhibitor. This conformation tie probably hinders the ability of AMP to enter its binding site. Second, PCIs preserved the conformation of a random-coil region (AK1_133~144_), thereby slowing down the transformation of AK1 from an open state to a closed state. If the chosen peptide of the AK1 segment (such as AK1_133~144_) had not been in the hinge region [35], strong interactions would have occurred between the protein and the MIP surface during the transformation from the open conformation to the closed conformation. While AK1 was integrating into the PCIs and the PCIs developed into conformation ties, the activities of AK1 revealed that such sequences must be rearranged during catalysis. Therefore, PCIs’ capture of enzymes creates a conformation tie and may also provide information about the motion of catalysis.

Table 3 summarizes the calculated kinetic parameters of the enzymatic catalysis. The kinetics of enzymes are generally governed by the Michaelis–Menten equation, as follows [40]:

(1) $$V=\frac{V_{max}\left[S\right]}{K_{m}+\left[S\right]}$$

where *V*, *V_max_*, [*S*], and *K*_m_ present the velocity, the substrate concentration, the maximum rate, and the Michaelis–Menten constant, respectively. Then, the turnover number (*K_cat_*) was determined using the following equation:

(2) $$K_{cat}=V_{max}/\left[E\right]$$

where [*E*] is the concentration of enzyme.

The results presented here show that, in terms of activity and catalytic efficiency, the PCIs performed best when AK1 was integrated. As can be seen in Table 3, enzymes catalyzed the reaction upon the PCIs’ capture and maintained higher catalytic activities.

In this study, the proposed MIP procedure involved construction of PCIs and recognition sites by adding templates in the special solvent systems of fabricated hinge-mediated MIPs on a QCM chip. Orädd et al. [42] also showed the closing and opening conformational transitions that characterize conformational dynamics during AK catalysis using time-resolved X-ray solution scattering (TR-XSS). Their results demonstrated that the conformational selection in the domain closure could be considered an induced-fit mechanism. Hetmann et al. [43] designed an AK-immobilized carbon nanomatrix with high and stable activity in the biocatalytic system, utilizing graphene oxide as the immobilization support. Compared with previous publications showing dynamic conformation transformation during AK1 catalysis, the resulting PCIs here had a unique proportion of random-coil, α-helix, or β-sheet cavities in the special solvents. This can be used to explore enzymes in relatively simple organic solvents and their mixtures [27,28,34]. The direct imprinting of an α-helical or β-sheet peptide via a special solvent led to the successful formation of PCIs. These imprints bound their mother protein in an aqueous solution. The ability of such PCIs to integrate into AK1 was correlated with exceptionally high selectivity in the enzyme. In addition, our peptide-imprinting method involves detailed inhibition mechanisms that can be deployed only when PCIs are being used. This method is suitable for shedding light on the relationship between the flexibility of each AK1 segment and the catalytic activity of enzymes. Nevertheless, PCIs are simpler and cheaper and can be used with the potential of natural bioenergetic repertoires. As far as we can discern, the current study represents the first time researchers have examined (1) the relationship between a hinge-imprinted cavity and the corresponding affinities of PCIs toward AK1, and (2) the linkage of PCIs to the catalytic function upon PCIs’ capture. These results suggest that this PCI technology is a valuable tool for studying the mechanisms of enzymes. Further study must be undertaken to investigate the effects of AK1 counteractions with PCIs in disease diagnosis. It will be an intriguing opportunity for tailor-made high-performance applications.

## 3. Materials and Methods

### 3.1. Synthesis of Templates

The synthetic peptides of the 15-mer AK1_9-23_ (KIIFVVGGPGSGKGT), AK1_37-51_ (RRIGQPTLLLYVDAG), AK1_69-83_ (LSTGDLLRSEVSSGS), and AK1_107-121_ (LETVLDMLRDAMVAK) were produced using the fluorenylmethoxy-carbonyl (Fmoc) method with a CEM Discover microwave-assisted peptide synthesizer (Kohan Co., Taipei, Taiwan), as described previously [34,44,45]. Subsequently, the purity of these peptides was monitored with an HPLC equipped with a reversed phase C18 column at a flow rate of 1 mL/min. The characteristic details regarding the HPLC chromatograms, mass spectra, and CD spectra of AK1_9-23_, AK1_37-51_, AK1_69-83_, and AK1_107-121_ are shown in the Supplementary Materials.

### 3.2. Preparation of Peptide Conformational Imprint Molecularly Imprinted Polymers on Quartz Crystal Microbalance Chips

The dual-electrode circular geometry of the QCM chips with a reproducibility frequency of 10 MHz was manufactured by screen printing onto commercially available AT-cut quartz plates (4.2 mm diameter; purchased from Tai-Yi Electronic Co., Taipei, Taiwan), as described before [44]. A 19 μM solution of (*N*-Acr-L-Cys-NHBn)_2_ with an aqueous mixture of 10 mL of HPLC-grade acetonitrile (ACN) and 0.1 mL of dimethylformamide (DMF) was prepared to immerse the QCM chips for 36 h, and they were then rinsed exhaustively with ACN and dried under vacuum.

A solution of 3 μmol of peptide templates (AK1_9-23_, AK1_37-51_, AK1_69-83_, or AK1_107-121_) dissolved in 0.3 mL of an aqueous solution (ACN/MB/TFE/buffer) and 55 μmol of *N*-Acr-L-His-NHBn (AHB), 55 μmol of acrylamide (Am), 110 μmol of *N*-acryltyramine (ATA), and 220 μmol of *N*,*N*-ethylene-bis-acrylamide (EBAA) was thoroughly mixed. After pipetting 3.5 μL of the aliquot on top of the gold electrode-(*N*-Acr-L-Cys-NHBn)_2_, the chip was placed into a 20 mL glass vial and irradiated with UV light at 350 nm for 6 h. A thin film on the gold surface was first immersed with 25 mM of urea solution containing 0.5% acetic acid (aq) and 0.5% Tween^®^ 20. These chips were then washed with deionized (DI) water and dried under vacuum. Finally, the pore structures were formed from different template molecules denoted AK1_9-23_ Cp, AK1_37-51_ Cp, AK1_69-83_ Cp, and AK1_107-121_ Cp, respectively.

### 3.3. The Quartz Crystal Microbalance System

All adsorption experiments were performed using not only a flow-injection system outfitted with an HPLC pump (flow rate 0.1 mL/min, Hitachi model L7110; Tokyo, Japan), but also a home-built flow cell, a sample injection valve (OMNIFIT model 1106, London, UK), a home-built oscillation circuit (including an oscillator and a frequency counter) (Linx Technology, Taipei, Taiwan), and a personal laptop (Acer Inc., Taipei, Taiwan). The polymer-coated quartz crystal microbalance chip was placed into the flow cell, and 100 μL of 5% acetic acid (aq) containing 25 mM urea solution was injected into the flow system to rapidly equilibrate the newly imprinted chips.

### 3.4. Determination of Binding Affinities of Peptide Conformational Imprint Molecularly Imprinted Polymers on Quartz Crystal Microbalance Chips

All adsorption experiments were performed using a quartz crystal microbalance system. For each measurement, 20 mM of PBS (pH 7.2) was first allowed to flow through the cell to obtain a stable baseline. Then, the template peptide or AK1 protein, with different concentrations, were injected into the cell. The binding of the AK1 templates or proteins to the MIP film caused a mass change, reflected in the resonance frequency.

### 3.5. Catalytic Activity of Free AK1 and AK1-Integrated QCM Chips Was Measured Using the HPLC Method

Catalytic activity of free AK1 and AK1-integrated QCM chips with 2 mL 20 mM phosphate buffer saline, pH 7.4, containing 25 μM AMP, 25 μM ATP, and 5 μM MgCl_2_, was measured at 37 °C for 90 min. The data were obtained using an HPLC system (Hitachi model L7110; Tokyo, Japan). Three consecutive measurements were used in the same chip. AK1 (11 nanograms) was purchased from Abcam^®^ (Cambridge, MA, USA).

## 4. Conclusions

In summary, this sensitive PCI device could become a useful tool for studying the mechanisms of enzymes. Specifically, it could help elucidate the motions of individual enzymatic regions during catalytic conversion. The results also indicate the importance of enzymes’ random-coil regions for the creation of flexibility during catalysis. Finally, we note that construction of an active site does not in itself guarantee the construction of a successful artificial enzyme; the conformation of an enzymatic system must be able to properly change during catalysis.

## Figures and Tables

**Table 1:** The chemical properties of peptides and their affinity toward MIP-QCM.

QCM Cp ^a^	Template	CD Conformation/ MIP Condition ^b^	K_d_ (nM)	Flow System ^b^
AK1_9-23_ Cp_AB_	KIIFVVGGPGSGKGT MW 1417 pI 9.2	RC/AB	5	AB
AK1_9-23_ Cp_MB_		Sheet/MB	7	MB
AK1_9-23_ Cp_3T7B_		Sheet/3T7B	10	MB
AK1_37-51_ Cp_AB_	LSTGDLLRSEVSSGS MW 1506 pI 4.2	RC/AB	4	AB
AK1_37-51_ Cp_MB_		Helix/MB	3	MB
AK1_37-51_ Cp_7T3B_		Helix/7T3B	5	MB
AK1_69-83_ Cp_AB_	LSTGDLLRSEVSSGS MW 1506 pI 4.2	RC/AB	3	AB
AK1_69-83_ Cp_MB_		Helix/MB	4	MB
AK1_69-83_ Cp_7T3B_		Helix/7T3B	3	MB
AK1_107-121_ Cp_2A8B_	RRIGQPTLLLYVDAG MW 1672 pI 8.2	RC/2A8B	3	AB
AK1_107-121_ Cp_7M3B_		Sheet/7M3B	6	MB
AK1_107-121_ Cp_3T7B_		Sheet/3T7B	2	MB
AK1_107-121_ Cp_7T3B_		Helix/7T3B	3	MB
AK1_133-144_ Cp_AB_	GETSGRVDNEE	RC/AB	<5	AB
AK1_141-155_ Cp_7M3B_	DNEETIKKRUETYYK	Helix/7M3B	<5	MB
AK1_157-171_ Cp_3T7B_	TEPVIAFYEKRGIVR	Sheet/3T7B	<5	MB
NIP	-	-	-	PBS

Cp, chip; CD, circular dichroism; NIP, non-imprinted polymer. ^a^ AHB/AM/ATA/EBAA = 1:1:2:4. AHB, *N*-Acr-L-His-NHBn; AM, acrylamide; ATA, *N*-acryltyramine; EBAA, *N*,*N*′-ethylenebisacrylamide. ^b^ RC, random coil; buffer: pH 7.2, 20 mM phosphate-buffered saline (PBS) buffer; AB, CH_3_CN/buffer = 1:1; 2A8B, CH_3_CN/buffer = 1:4; MB, CH_3_OH/buffer = 1:1; 3M7B, CH_3_OH:buffer = 3:7; 7M3B, CH_3_OH:buffer = 7:3; 3T7B, TFE/buffer = 3:7; 7T3B, TFE/buffer = 7:3.

**Table 2:** The effect of integrating a conformation tie into AK1’s catalytic activity.

QCM Cp	CD Structure	K_d_ (pM)	Conversion ^a^ (%)	Activity ^b^ (nM⋅min^−1^⋅ng^−1^)
AK1_9-23_ Cp_AB_	RC	-	-	-
AK1_9-23_ Cp_MB_	Sheet	9000	0.2	0.051
AK1_9-23_ Cp_3T7B_	Sheet	50	0.39	0.099
AK1_37-51_ Cp_AB_	RC	3000	41.42	10.5
AK1_37-51_ Cp_MB_	Helix	1000	41.58	10.5
AK1_37-51_ Cp_7T3B_	Helix	70	42.36	10.7
AK1_69-83_ Cp_AB_	RC	2000	41.82	10.6
AK1_69-83_ Cp_MB_	Helix	400	42.49	10.7
AK1_69-83_ Cp_7T3B_	Helix	40	43.35	10.9
AK1_107-121_ Cp_2A8B_	RC	200	42.21	10.7
AK1_107-121_ Cp_7M3B_	Sheet	50	42.61	10.8
AK1_107-121_ Cp_3T7B_	Sheet	60	42.55	10.7
AK1_107-121_ Cp_7T3B_	Helix	200	42.20	10.7
AK1_133-144_ Cp_AB_	RC	70	0.13	0.033
AK1_141-155_ Cp_7M3B_	Helix	50	42.85	10.8
AK1_157-171_ Cp_3T7B_	Sheet	60	42.42	10.7
NIP ^c^	-	-	0.32	0.081
Free AK1 ^d^	-	-	12.01	3.03

Cp, chip; CD, circular dichroism; NIP, non-imprinted polymer. Assays were performed in the presence of an AK1-integrated QCM Cp (~20 Hz) with 2 mL 20 mM PBS (Na_2_HPO_4_ and NaCl), pH 7.4, containing 25 µM AMP, 25 µM ATP, and 5 μM MgCl_2_ at 37 °C for 90 min. ^a^ Conversion (%) as a measure of AK1 activity was determined by the rates of AMP and ATP transphosphorylation. The data were obtained using HPLC. ^b^ The mean values of three consecutive measurements using the same chip. ^c^ Only one measurement was performed. ^d^ AK1 (11 nanograms; purchased from Abcam^®^, Cambridge, MA, USA).

**Table 3:** Summary of the calculated kinetic parameters of catalytic enzymes.

QCM Cp	*V*^a^ M⋅S^−1^	*V*_max_ M⋅S^−1^	*K*_m_ M	*K*_cat_ S^−1^	*K*_cat_/*K*_m_ M^−1^⋅S^−1^
AK1_9-23_ Cp_AB_	-	-	-	-	-
AK1_9-23_ Cp_MB_	6.55 × 10^−11^	3.36 × 10^−11^	2.57 × 10^−5^	0.007	283.55
AK1_9-23_ Cp_3T7B_	1.01 × 10^−10^	9.33 × 10^−9^	4.62 × 10^−3^	2.02	437.23
AK1_37-51_ Cp_AB_	1.58 × 10^−10^	2.43 × 10^−10^	7.70 × 10^−5^	0.053	683.98
AK1_37-51_ Cp_MB_	1.60 × 10^−10^	7.39 × 10^−10^	2.31 × 10^−4^	0.16	692.64
AK1_37-51_ Cp_7T3B_	4.83 × 10^−10^	3.19 × 10^−8^	3.30 × 10^−3^	6.90	2090.91
AK1_69-83_ Cp_AB_	1.08 × 10^−10^	2.49 × 10^−10^	1.16 × 10^−4^	0.054	467.53
AK1_69-83_ Cp_MB_	8.21 × 10^−10^	9.48 × 10^−9^	5.78 × 10^−4^	2.053	3554.11
AK1_69-83_ Cp_7T3B_	1.22 × 10^−9^	1.41 × 10^−7^	5.77 × 10^−3^	30.50	5281.38
AK1_107-121_ Cp_2A8B_	1.20 × 10^−10^	2.77 × 10^−9^	1.12 × 10^−3^	0.60	519.48
AK1_107-121_ Cp_7M3B_	5.42 × 10^−10^	5.01 × 10^−8^	4.62 × 10^−3^	10.84	2346.32
AK1_107-121_ Cp_3T7B_	3.12 × 10^−10^	2.40 × 10^−8^	3.85 × 10^−3^	5.20	1350.65
AK1_107-121_ Cp_7T3B_	1.42 × 10^−10^	3.28 × 10^−9^	1.12 × 10^−3^	0.71	614.72
NIP ^b^	6.52 × 10^−11^	-	-	-	-
Free AK1 ^c^	1.29 × 10^−11^	-	-	-	-

Cp, chip; NIP, non-imprinted polymer. ^a^ The data were obtained using HPLC. ^b^ Only one measurement was performed. ^c^ AK1 (11 nanograms; purchased from Abcam^®^, Cambridge, MA, USA).

## Data Availability

Not applicable.

## Supplementary Materials

The following supporting information can be downloaded at: https://www.mdpi.com/article/10.3390/ijms23126521/s1.
