# Supplementary material for: Integration of Adenylate Kinase 1 with Its Peptide Conformational Imprint
Source: Int J Mol Sci. 2022 Jun 10;23(12):6521. doi: 10.3390/ijms23126521 (PMC9223553; doi:10.3390/ijms23126521)
Supplement: Supplementary file 1 [file ijms-23-06521-s001.zip › ijms-1727220-supplementary.pdf]

Supporting information

Integration of adenylate kinase 1 with its peptide conformational imprint

Cheng-Hsin Wu [1], Chung-Yin Lin [2,3,*], Tzu-Chieh Lin [1], and Dar-Fu Tai [1,*]

[1] Department of Chemistry, National Dong Hwa University, Hualien 97403, Taiwan; m9812022@gms.ndhu.edu.tw (C-H.W.); m9812012@gms.ndhu.edu.tw (T-C.L.); dftai@gms.ndhu.edu.tw (D-F.T.)

[2] Medical Imaging Research Center, Institute for Radiological Research, Chang Gung University, Taoyuan 33302, Taiwan; winwood7@mail.cgu.edu.tw (C-Y.L.)

[3] Department of Nephrology and Clinical Poison Center, Chang Gung Memorial Hospital, Taoyuan 33302, Taiwan

*Correspondence: winwood7@mail.cgu.edu.tw (C-Y.L.); dftai@gms.ndhu.edu.tw (D-F.T.); Tel.: +886-3-211-8800 (ext. 3865) (C-Y.L.)

Results and Discussion

Synthesis and properties of the 15-mer AK1 templates

Characterization of AK1$_{9\text{-}23}$ (KIIFVVGGPGSGKGT)

The HPLC chromatogram **revealed a sharp AK1$_{9\text{-}23}$ peak at a retention time of ~3.3 min and purity was calculated to be ~81% as compared to standards** (Figure S1). The sequence being KIIFVVGGPGSGKGT has a reported m/z value of AK1$_{9\text{-}23}$ at **1,417.73** g·mol^{-1} (Figure S2). The CD analysis of synthetic AK1$_{9\text{-}23}$ segment in aqueous solution showed a typical spectrum of disordered structure (Figure S3). The proportion of the sequence **assumed** random-coil or β-sheet secondary structure in the different solvent environments, showing a general predominance of random- coil and β-sheet conformation.

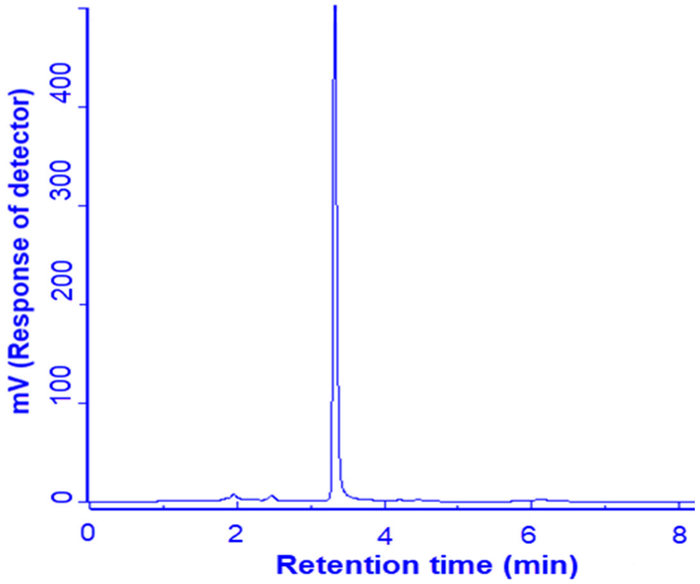

Figure S1. HPLC chromatogram of AK1$_{9\text{-}23}$ (KIIFVVGGPGSGKGT).

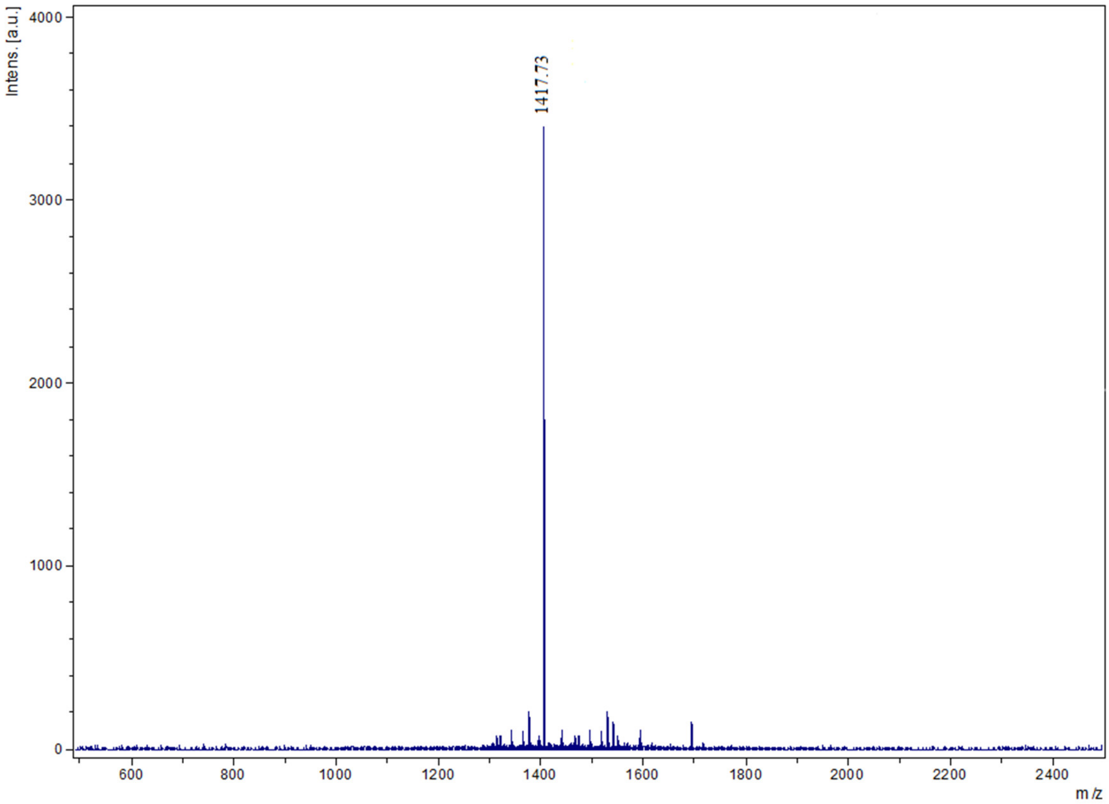

Figure S2. Mass spectrum of AK1$_{9\text{-}23}$ (KIIFVVGGPGSGKGT).

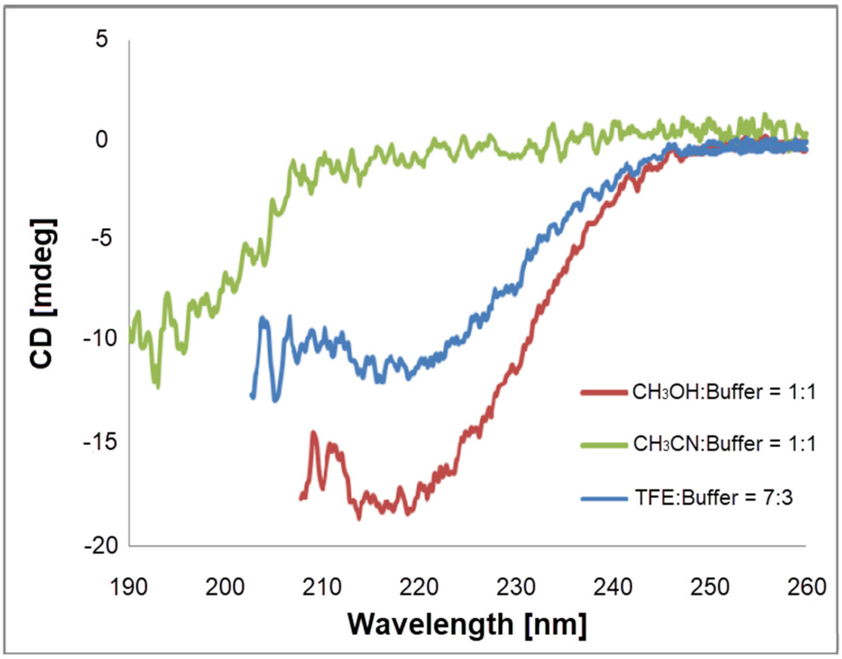

Figure S3. Signature CD spectra demonstrating the effect of different solvent systems on the secondary structure of the AK1$_{9\text{-}23}$ (KIIFVVGGPGSGKGT).

Characterization of AK1$_{37-51}$ (LSTGDLLRSEVSSGS)

Subsequently, the HPLC chromatogram **revealed a sharp AK1$_{37-51}$ peak at a retention time of ~2.4 min and purity was calculated to be ~72% as compared to standards** (Figure S4). The reported m/z value of AK1$_{37-51}$ was observed **at 1,507.63 g·mol^{-1}** (Figure S5). The proportion of the sequence **was assumed** random-coil or α-helix secondary structure in the different solvent environments, showing a general predominance of random-coil and α-helix conformation (Figure S6).

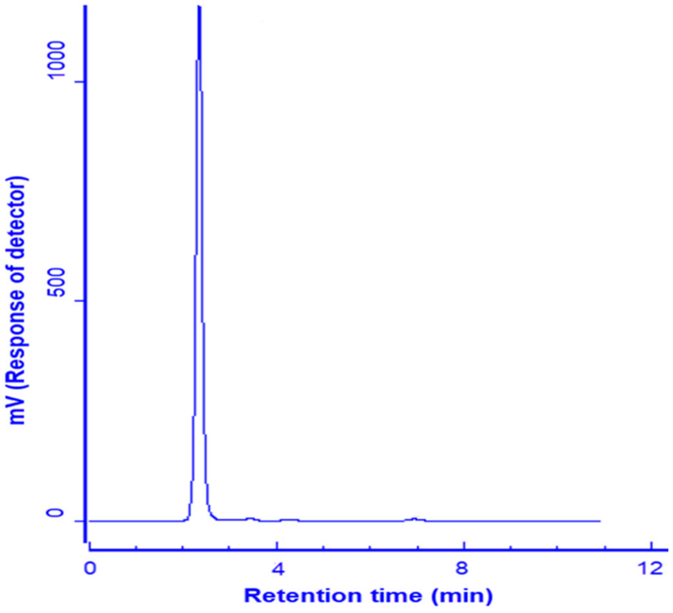

Figure S4. HPLC chromatogram of AK1$_{37-51}$ (LSTGDLLRSEVSSGS).

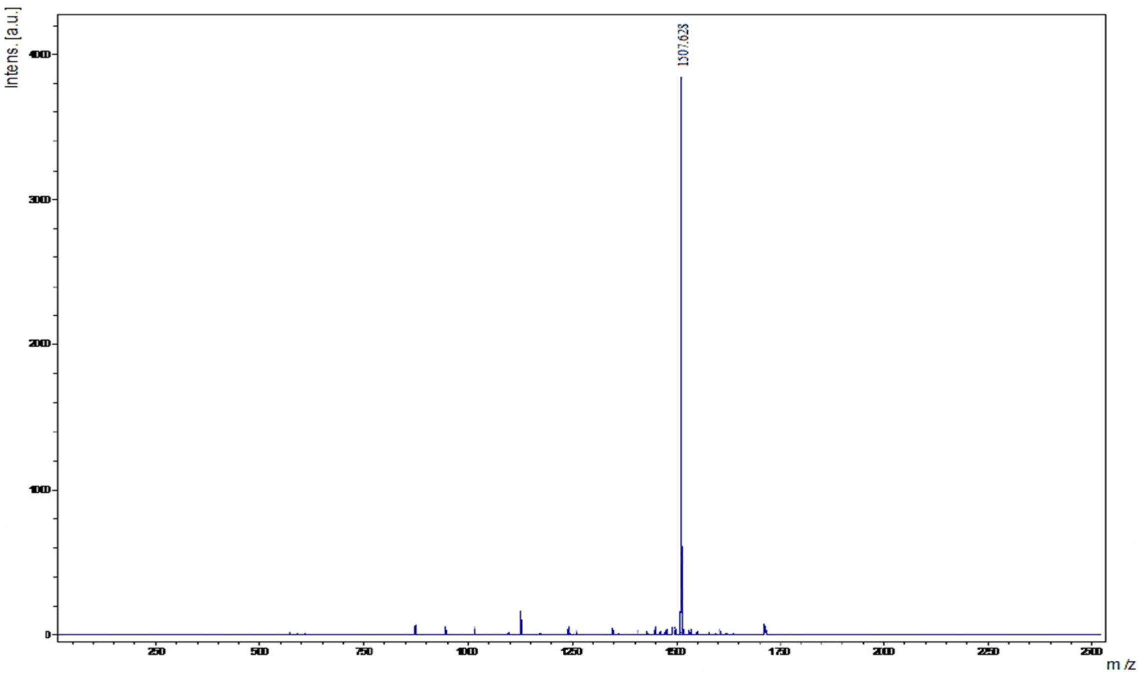

Figure S5. Mass spectrum of AK1$_{37-51}$ (LSTGDLLRSEVSSGS).

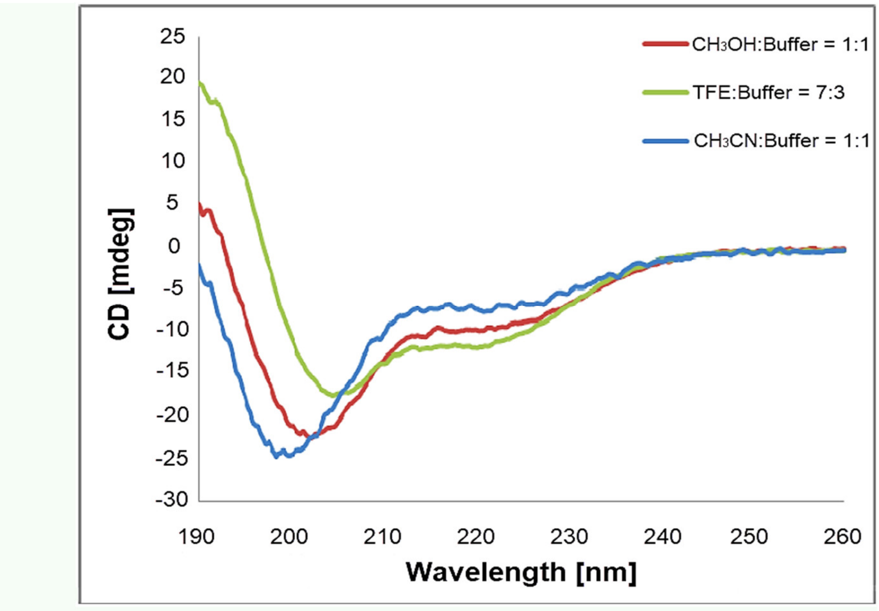

Figure S6. Signature CD spectra demonstrating the effect of different solvent systems on the secondary structure of the $AK1_{37\text{-}51}$ (LSTGDLLRSEVSSGS).

Characterization of $AK1_{69\text{-}83}$ (LETVLDMLRDAMVAK)

Next, the purity of $AK1_{69\text{-}83}$ was monitored by HPLC equipped with an RP-18 at a flow rate of 1 mL/min. Sharp peak was observed at a retention time of ~3.5 min (Figure S7); **this peptide's** purity was **calculated** to be ~80% **as compared to standards**. The reported m/z value of $AK1_{69\text{-}83}$ was observed at **1,705.07 g·mol^{-1}** (Figure S8). The proportion of the sequence **assumed** random-coil or **α-**helix secondary structure in the different solvent environments, showing a general predominance of random-coil and α-helix conformation (Figure S9).

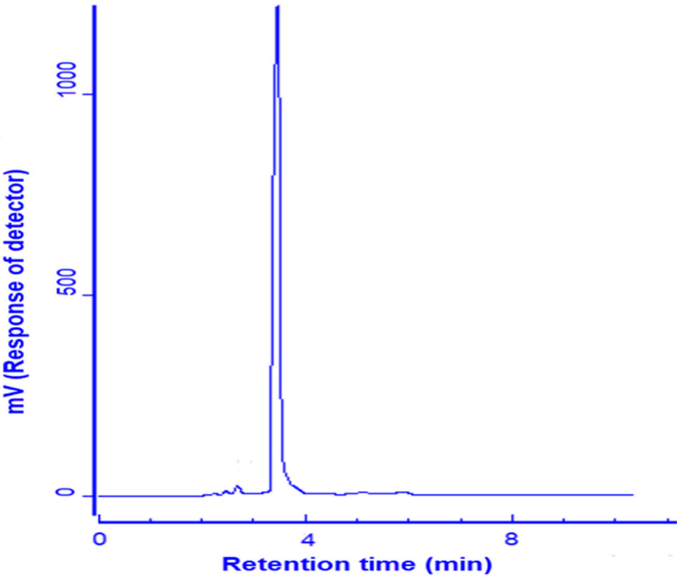

Figure S7. HPLC chromatogram of $AK1_{69\text{-}83}$ (LETVLDMLRDAMVAK).

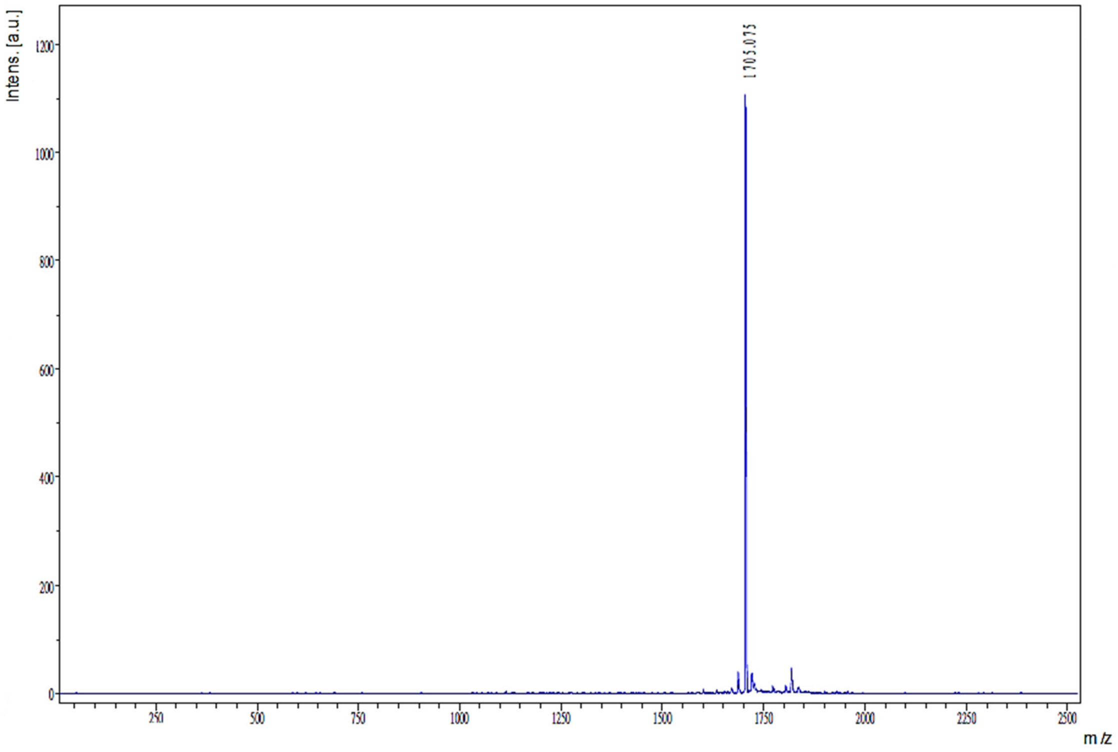

Figure S8. Mass spectrum of AK1$_{69-83}$ (LETVLDMLRDAMVAK).

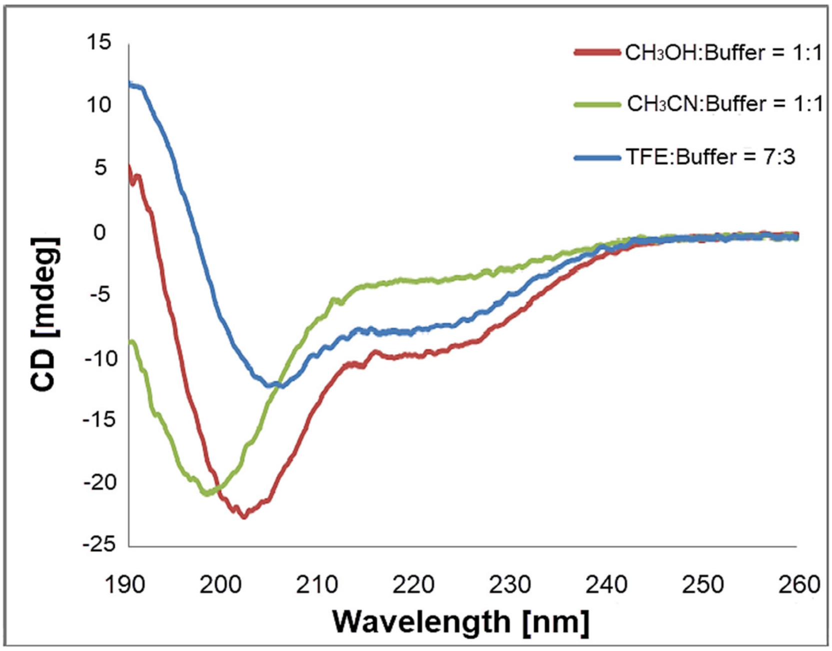

Figure S9. Signature CD spectra demonstrating the effect of different solvent systems on the secondary structure of the AK1$_{69-83}$ (LETVLDMLRDAMVAK).

Characterization of AK1$_{107-121}$ (RRIGQPTLLLYVDAG)

Then, the HPLC chromatogram **revealed a sharp AK1$_{107-121}$ peak at a retention time of ~2.1 min and purity was calculated to be ~78% as compared to standards** (Figure S10). The reported m/z value of AK1$_{107-121}$ was observed at **1,671.92 g·mol^{-1}** (Figure S11). The CD analysis of synthetic AK1$_{107-121}$ segment in aqueous solution showed a typical spectrum of disordered structure. **The 70% TFE used in the special solvent system exhibited spectral deviation from β-sheet to α-helix conformation when compared to 30% TFE used in this system** (Figure S12). The proportion of the sequence was assumed α-helical, β-sheet, or random-coil secondary structure in the different environments. The results show a general predominance of random-coil, α-helix, and β-sheet conformation.

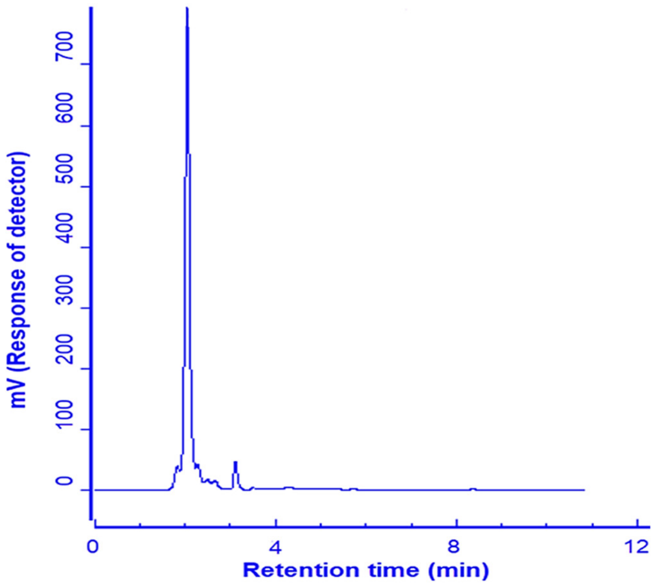

Figure S10. HPLC chromatogram of AK1$_{107-121}$ (RRIGQPTLLLYVDAG).

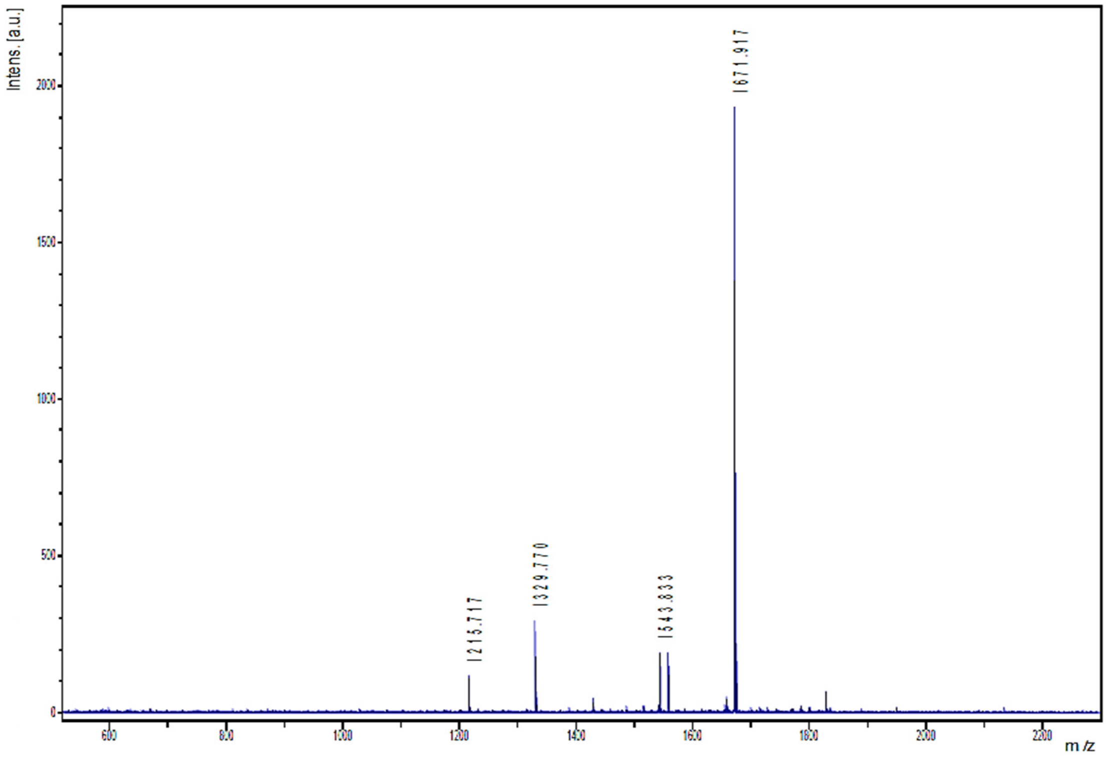

Figure S11. Mass spectrum of AK1$_{107\text{-}121}$ (RRIGQPTLLLYVDAG).

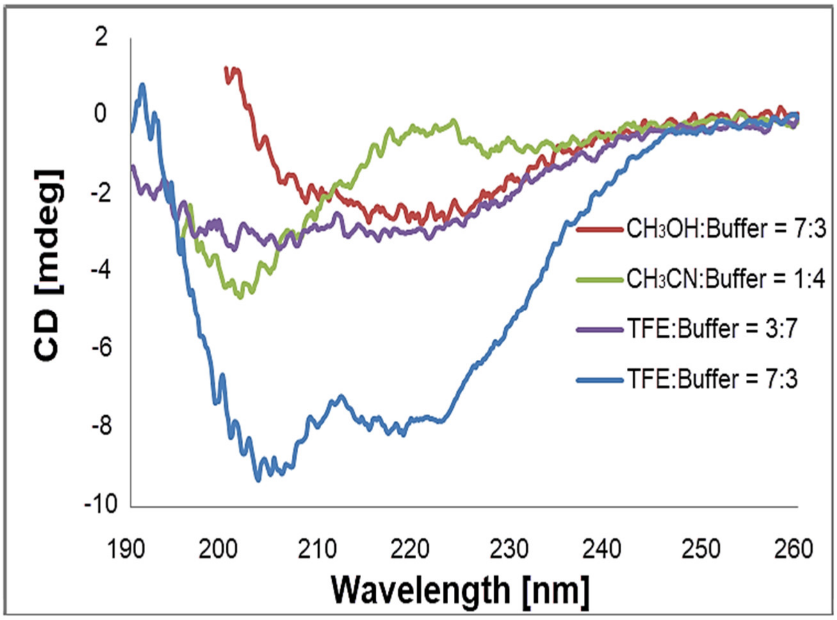

Figure S12. Signature CD spectra demonstrating the effect of different solvent systems on the secondary structure of the AK1$_{107\text{-}121}$ (RRIGQPTLLLYVDAG).
